# An analysis of the global, regional, and national burden of blindness and vision loss between 1990 and 2021: the findings of the Global Burden of Disease Study 2021

**DOI:** 10.3389/fpubh.2025.1560449

**Published:** 2025-04-29

**Authors:** Lijuan Que, Qin Zhu, Chun Jiang, Qianyi Lu

**Affiliations:** ^1^Department of Ophthalmology, The First Affiliated Hospital of Soochow University, Suzhou, China; ^2^Department of Ophthalmology, Second Affiliated Hospital of Anhui University of Traditional Chinese Medicine, Hefei, Anhui, China; ^3^Department of Ophthalmology, Lu’an People’s Hospital, Lu’an, Anhui, China

**Keywords:** blindness, vision loss, years lived with disability, prevalence, age-standardized rates

## Abstract

**Objectives:**

To evaluate the global burden of blindness and vision loss (BVL) from 1990 to 2021 using a retrospective analysis of epidemiological data from Global Burden of Disease (GBD) study 2021 and to project trends over the next 15 years.

**Methods:**

This retrospective study analyzed data on years lived with disability (YLDs) and prevalence across 204 countries and territories. Trends over time were assessed using estimated annual percentage change (EAPC) of the age-standardized rates (ASR), while decomposition analysis quantified the contributions of population aging, growth, and epidemiological shifts. Socioeconomic disparities were evaluated using the Slope Index of Inequality and the Concentration Index. Future trends in age-standardized prevalence rates (ASPR) and age-standardized YLDs rates (ASYR) were projected using Bayesian age–period–cohort modeling.

**Results:**

Between 1990 and 2021, global YLDs due to BVL more than doubled, increasing from 14.31 to 29.16 million. The prevalence of BVL surged by 246.8%, with a disproportionately higher burden observed among females. Regions with low to middle Socio-Demographic Index (SDI) scores exhibited the highest ASPR and ASYR. Population growth and aging were the primary drivers of the increasing burden, while epidemiological trends had mixed effects. Significant socioeconomic disparities persist, with a higher concentration of BVL burden in economically disadvantaged regions. Projections indicate a continued increase in BVL burden through 2036, particularly among women and older populations.

**Conclusion:**

Despite advancements in healthcare, the global burden of BVL has substantially increased over the past 32 years, driven by demographic and socioeconomic factors. Persistent disparities highlight the urgent need for targeted public health interventions, equitable resource allocation, and policy initiatives to address the growing impact of BVL worldwide.

## Introduction

With socioeconomic development, advancements in medical science and technology, and increasing life expectancy, blindness and vision loss (BVL) have become major global public health and socioeconomic challenges. According to recent estimates, approximately 43.3 million people worldwide are affected by blindness, while 295 million experience moderate to severe vision impairment. These numbers are projected to rise to 61 million and 834 million, respectively, by 2050 (1). The World Health Organization (WHO) has emphasized the substantial healthcare and economic burden associated with BVL, estimating global economic losses at $411 billion annually, which imposes significant strain on healthcare systems and government budgets (2).

To address the growing challenge of vision impairment, the WHO launched the Global Elimination of Avoidable Blindness and Visual Impairment initiative (3). This program has led to significant improvements, with a reported downward trend in the burden of moderate-to-severe visual impairment. However, despite these efforts, BVL remains a persistent public health concern, and projections indicate that its burden will continue to rise in the coming decades. Given the widespread impact of BVL, further in-depth research is urgently needed (2). Globally, the burden of BVL varies significantly across countries, influenced by factors such as ethnicity, income levels, and social determinants of health (4, 5).

Previous studies, utilizing data from the Global Burden of Disease (GBD) 2019, provided an initial assessment of the global BVL burden (6). Based on the latest GBD 2021 data, this study systematically examined the trends in prevalence and years lived with disability (YLDs) due to BVL across different countries and Socio-Demographic Index (SDI) levels from 1990 to 2021. Building on this foundation, we further analyzed the temporal trends of age-standardized YLDs rate (ASYR) and age-standardized prevalence rate (ASPR) of BVL using the Estimated Annual Percentage Change (EAPC), in conjunction with 95% uncertainty intervals (UI) and 95% confidence intervals (CI). Additionally, this study projected potential changes in the global BVL burden over the next 15 years (2021–2036). These findings will provide valuable insights for policymakers, enabling more effective allocation of healthcare resources and the development of targeted public health interventions to mitigate the burden of BVL worldwide.

## Methods

### Data

This study utilized the latest available data from the Global Health Data Exchange (GHDx),^1^ which provides comprehensive information from the GBD 2021 dataset. The GBD employs standard methodologies to quantify health outcomes and risk factors across 459 conditions, including vision loss from conditions such as glaucoma, cataracts, macular degeneration, and other eye disorders not separately classified (7). This data enables robust assessments of global disease burden by providing metrics such as prevalence and YLDs.

To address potential data limitations and variability, the GBD employs a meta-regression framework, ensuring reliable and consistent metrics for evaluating disease burden. We specifically extracted data on YLDs and the prevalence of BVL for the period 1990 to 2021.

In addition, we incorporated the SDI, a composite metric that accounts for education, economic development, and fertility rates. Regions were stratified into five quintiles (high, high-middle, middle, low-middle, and low) based on their SDI scores.

### Factor calculations

The EAPC metric was calculated by performing linear regression of the natural logarithm of ASYR and ASPR on the year of record (8). The age-standardized rate (ASR) was obtained by the formula:


$$ASR\;\left(\mathrm{per}\;100,000\;\mathrm{population}\right)=\frac{\sum \frac{A}{i=1}\mathrm{aiwi}}{\sum \frac{A}{i=1}wi}\times 100,000$$

The natural logarithm of the ASR was modeled using a linear regression equation: y = *α* + *β*x + *ε*, where y = ln (ASR), and x represents the calendar year. The EAPC was subsequently calculated using the formula: EAPC = 100 × (exp(β) − 1) (9). A positive EAPC (> 0) indicated an increasing trend, a negative EAPC (< 0) indicated a decreasing trend, and an EAPC of 0 indicated stability.

To further understand the factors influencing the global BVL burden, we performed a decomposition analysis (10). This analysis partitioned the BVL burden into three main components: population size, population age distribution, and prevalence. By isolating the contributions of demographic shifts and disease-related factors, we gained a clearer understanding of the influence of aging, population growth, and epidemiological changes on BVL trends.

We conducted frontier analysis to assess the relationship between a region’s sociodemographic progress and the achievable reduction in disease burden (11). This method estimates a nonlinear frontier representing the minimum BVL burden a region can achieve based on its current development status. Using non-parametric data envelopment analysis, we compared the actual YLDs rate of each country with the expected YLDs rate based on its SDI score. The resulting effective difference provides insight into the potential health gains achievable if the region reached its development frontier.

To assess income-based health inequalities, we analyzed the distribution of YLDs and ASYR across different SDI quintiles (12). Following WHO guidelines, we employed the Slope Index of Inequality (SII) and Concentration Index (CI) to measure both absolute and relative disparities in the BVL burden across countries.

We used Bayesian age–period–cohort (BAPC) modeling, implemented in R using the BAPC and INLA software packages, to project future trends in ASYR and ASPR for BVL from 2022 to 2036 (13). The projections were stratified by gender and region, providing a forward-looking perspective on global BVL trends.

All statistical analyses and visualizations were conducted using R version 4.4.1. A *p*-value of < 0.05 was considered statistically significant for all analyses.

## Results

### Overview of the global burden of BVL

**Figure 1:**
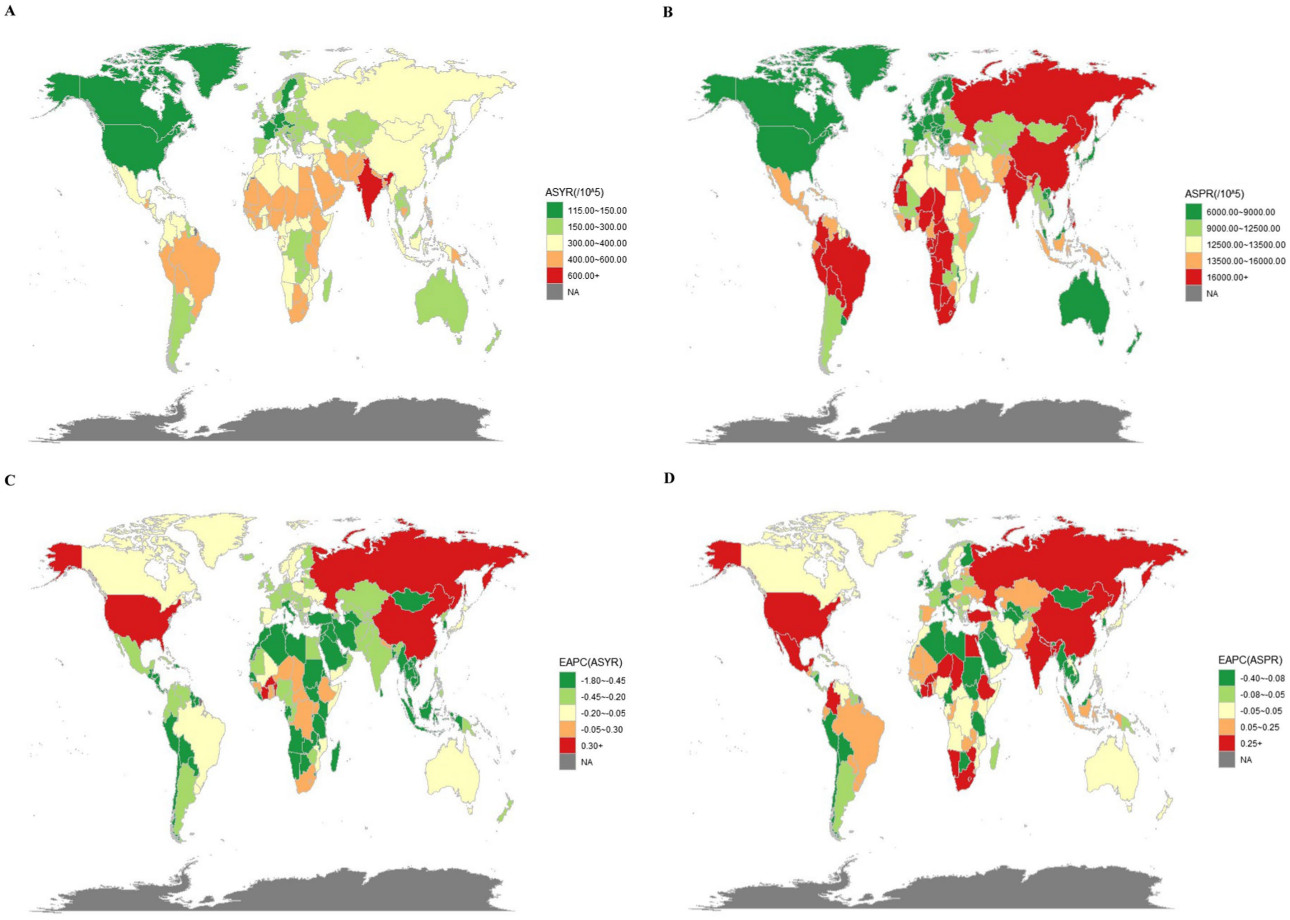
The YLDs and prevalence of BVL in 204 countries and territories. **(A)** The ASYR of BVL in 2021. **(B)** The ASPR of BVL in 2021. **(C)** The EAPC of ASYR of BVL from 1990 to 2021. **(D)** The EAPC of ASPR of BVL from 1990 to 2021.

Figure 1 presents the age-standardized years lived with disability (ASYR) and age-standardized prevalence rate (ASPR) for blindness and vision loss (BVL) across 204 countries and territories. The global YLDs due to BVL more than doubled, increasing from 14.31 million (95% UI: 9.81–20.34) in 1990 to 29.16 million (95% UI: 19.03–42.92) in 2021 (Table 1). Over the same period, the ASYR increased from 342.01 (95% UI: 237.87–482.17) in 1990 to 342.78 (95% UI: 224.21–503.61) in 2021, with an estimated annual percentage change (EAPC) of 0.14 (95% CI: 0.08–0.19). In 2021, the number of YLDs in females (16.17 million, 95% UI: 10.61–23.62) was higher than in males (13.00 million, 95% UI: 8.42–19.25), and the ASYR showed a faster increase in females than in males (Supplementary Figure 1).

**Table 1:** YLDs of BVL in 1990 and 2021 for both sexes and all locations, with EAPC from 1990 to 2021.

Location	1990		2021		1990–2021
	YLDs No. × 10^6^(95%UI)	ASYR(95%UI)	YLDs No. × 10^6^(95%UI)	ASYR(95%UI)	EAPC (95%CI)
Global	14.31 (9.81, 20.34)	342.01 (237.87, 482.17)	29.16 (19.03, 42.92)	342.78 (224.21, 503.61)	0.14 (0.08 to 0.19)
Sex					
Female	7.88 (5.42, 11.21)	354.94 (245.57, 504.92)	16.17 (10.61, 23.62)	363.56 (237.52, 533.39)	0.22 (0.16 to 0.28)
Male	6.44 (4.41, 9.13)	329.47 (228.62, 463.42)	13.00 (8.42, 19.25)	320.54 (209.71, 471.8)	0.14 (0.08 to 0.19)
SDI					
High SDI	1.69 (1.10, 2.51)	166.06 (107.07, 246.44)	2.75 (1.77, 4.16)	164.31 (102.75, 249.44)	0.04 (0.01 to 0.08)
High-middle SDI	2.79 (1.87, 4.04)	282.18 (193.2, 408.21)	5.36 (3.41, 8.22)	293.72 (186.7, 449.95)	0.28 (0.21 to 0.35)
Middle SDI	4.59 (3.15, 6.49)	413.76 (289.32, 580.14)	10.55 (6.85, 15.62)	396.12 (260.36, 577.94)	−0.01 (−0.09 to 0.06)
Low-middle SDI	3.95 (2.77, 5.48)	606.86 (434.81, 833.54)	7.70 (5.13, 11.02)	509.85 (347.87, 719.48)	−0.46 (−0.51 to −0.41)
Low SDI	1.29 (0.89, 1.81)	524.07 (374.66, 719.54)	2.79 (1.85, 4.03)	473.57 (326.3, 660.44)	−0.24 (−0.29 to −0.2)
Region					
Andean Latin America	0.12 (0.08, 0.17)	494.54 (340.78, 704.04)	0.25 (0.16, 0.36)	400.19 (269.29, 580.72)	−0.77 (−0.8 to −0.75)
Australasia	0.04 (0.02, 0.05)	162.9 (102.74, 243.55)	0.07 (0.04, 0.10)	155.97 (96.85, 237.29)	−0.11 (−0.13 to −0.09)
Caribbean	0.10 (0.07, 0.14)	346.01 (237.45, 497.86)	0.16 (0.10, 0.23)	298.15 (197.79, 437.74)	−0.45 (−0.47 to −0.44)
Central Asia	0.16 (0.11, 0.23)	328.82 (228.87, 472.45)	0.24 (0.16, 0.35)	294.15 (201.84, 427.41)	−0.36 (−0.38 to −0.34)
Central Europe	0.24 (0.16, 0.36)	171.24 (111.87, 254.63)	0.31 (0.20, 0.46)	159.17 (101.94, 240.13)	−0.23 (−0.25 to −0.21)
Central Latin America	0.40 (0.27, 0.56)	400.32 (282.1, 561.62)	0.87 (0.58, 1.27)	343.83 (230.44, 503.65)	−0.38 (−0.44 to −0.32)
Central Sub-Saharan Africa	0.10 (0.06, 0.16)	335.65 (205.97, 532.41)	0.25 (0.14, 0.41)	324.03 (195.47, 518.98)	−0.08 (−0.11 to −0.05)
East Asia	2.63 (1.76, 3.85)	293.43 (201.14, 427.4)	6.63 (4.11, 10.49)	318.92 (200.39, 497.18)	0.43 (0.27 to 0.58)
Eastern Europe	0.82 (0.54, 1.21)	310.86 (204.43, 458.72)	0.98 (0.61, 1.51)	312.36 (194.32, 483)	0.31 (0.2 to 0.42)
Eastern Sub-Saharan Africa	0.36 (0.25, 0.50)	421.46 (301.34, 580.71)	0.76 (0.52, 1.08)	374.77 (263.77, 518.96)	−0.32 (−0.34 to −0.29)
High-income Asia Pacific	0.32 (0.20, 0.49)	167 (106.14, 249.49)	0.52 (0.34, 0.78)	155.97 (97.08, 238.95)	−0.2 (−0.21 to −0.18)
High-income North America	0.42 (0.27, 0.63)	133.17 (84.24, 202.17)	0.69 (0.44, 1.05)	134.11 (83.8, 206.86)	0.3 (0.2 to 0.41)
North Africa and Middle East	0.91 (0.64, 1.25)	471.16 (338.64, 647.39)	1.88 (1.28, 2.72)	386.78 (270.54, 545.75)	−0.69 (−0.71 to −0.66)
Oceania	0.01 (0.009, 0.02)	419.15 (294, 593.77)	0.03 (0.02, 0.04)	374.47 (256.98, 538.13)	−0.36 (−0.43 to −0.3)
South Asia	4.25 (3.00, 5.91)	705.16 (506.27, 962.9)	9.35 (6.14, 13.55)	605.29 (407, 862.35)	−0.34 (−0.41 to −0.27)
Southeast Asia	1.24 (0.86, 1.74)	453.08 (321.23, 626.13)	2.35 (1.59, 3.41)	361.6 (252.27, 517.68)	−0.79 (−0.82 to −0.76)
Southern Latin America	0.10 (0.07, 0.15)	220.17 (145.61, 320.1)	0.16 (0.10, 2.34)	198.25 (127.42, 292.54)	−0.31 (−0.33 to −0.3)
Southern Sub-Saharan Africa	0.17 (0.11, 0.26)	523.31 (344.11, 781.52)	0.31 (0.19, 0.50)	473.88 (299.35, 723)	−0.07 (−0.16 to 0.02)
Tropical Latin America	0.50 (0.34, 0.72)	474.38 (328.07, 673.82)	1.02 (0.68, 1.48)	405.41 (271.52, 592.24)	−0.17 (−0.31 to −0.04)
Western Europe	0.97 (0.66, 1.41)	191.28 (128.71, 278.3)	1.29 (0.88, 1.87)	174.14 (114.36, 257.08)	−0.28 (−0.3 to −0.26)
Western Sub-Saharan Africa	0.47 (0.32, 0.66)	474.83 (337.31, 660.5)	1.07 (0.72, 1.54)	449.82 (315.5, 631.6)	−0.21 (−0.26 to −0.16)

UI, uncertainty interval; CI, confidence interval; EAPC, estimated annual percentage change; SDI, socio-demographic index; YLDs, years lived with disability; ASYR, age-standardized YLDs rates.

The total number of cases in 2021 was 246.8% higher than in 1990. The ASPR rose from 12.5 thousand (95% UI: 10.3–15.2) in 1990 to 15.8 thousand (95% UI: 12.8–19.5) in 2021, with an EAPC of 1.09 (95% CI: 0.97–1.2). The growth rates for females and males were similar, as shown in Supplementary Table 1 and Figure 1.

### Burden of BVL by SDI quintiles

We analyzed YLDs and prevalence by SDI quintiles from 1990 to 2021 (Table 1; Supplementary Table 1). In 2021, the middle-SDI region accounted for the highest number of YLDs (10.55 million; 95% UI: 6.85–15.62) and cases (495.40 million; 95% UI: 395.98–617.93). Regarding age-standardized rates, the low-middle SDI quintiles showed the highest ASYR (509.85, 95% UI: 347.87–719.48) and ASPR (19.4 thousand, 95% UI: 16.0–23.6) in 2021.

In terms of EAPC, high-middle SDI regions saw the most significant increase in YLDs (0.28, 95% CI: 0.21–0.35), and regions with middle SDI experienced the largest increase in prevalence (1.11, 95% CI: 0.99–1.23) from 1990 to 2021.

### Regional differences in BVL burden

In 2021, South Asia exhibited the highest YLDs for BVL, with 9.35 million (95% UI: 6.14–13.55) and the highest ASYR (605.29, 95% UI: 407–862.35). Southeast Asia showed the slowest growth in YLDs, with an EAPC of −0.79 (95% CI: −0.82 to −0.76) from 1990 to 2021 (Table 1). South Asia also had the highest prevalence (402.13 million, 95% UI: 319.62–496.50). Southern Sub-Saharan Africa reported the highest ASPR (26.6 thousand, 95% UI: 21.4–33.0). Over the 32-year period, South Asia showed the greatest increase in prevalence (EAPC = 1.51, 95% CI: 1.29–1.72) (Supplementary Table 1).

ASYR and ASPR displayed an inverted “V” relationship with SDI, with both rates increasing gradually when SDI < 0.4 but decreasing sharply after SDI > 0.4 (Supplementary Figure 2). The burden of BVL in China in 2021 exceeded the global average (Supplementary Figure 3).

### Decomposition analysis on YLDs and prevalence

Over the past 32 years, the global prevalence and YLDs for BVL have risen significantly. The decomposition analysis revealed that aging, population growth, and epidemiological changes contributed −138.52, 334.37%, and −95.85%, respectively, to the increase in YLDs. In contrast, aging, population growth, and epidemiological changes accounted for 30.04, 43.13, and 26.83% of the increase in prevalence, respectively.

**Figure 2:**
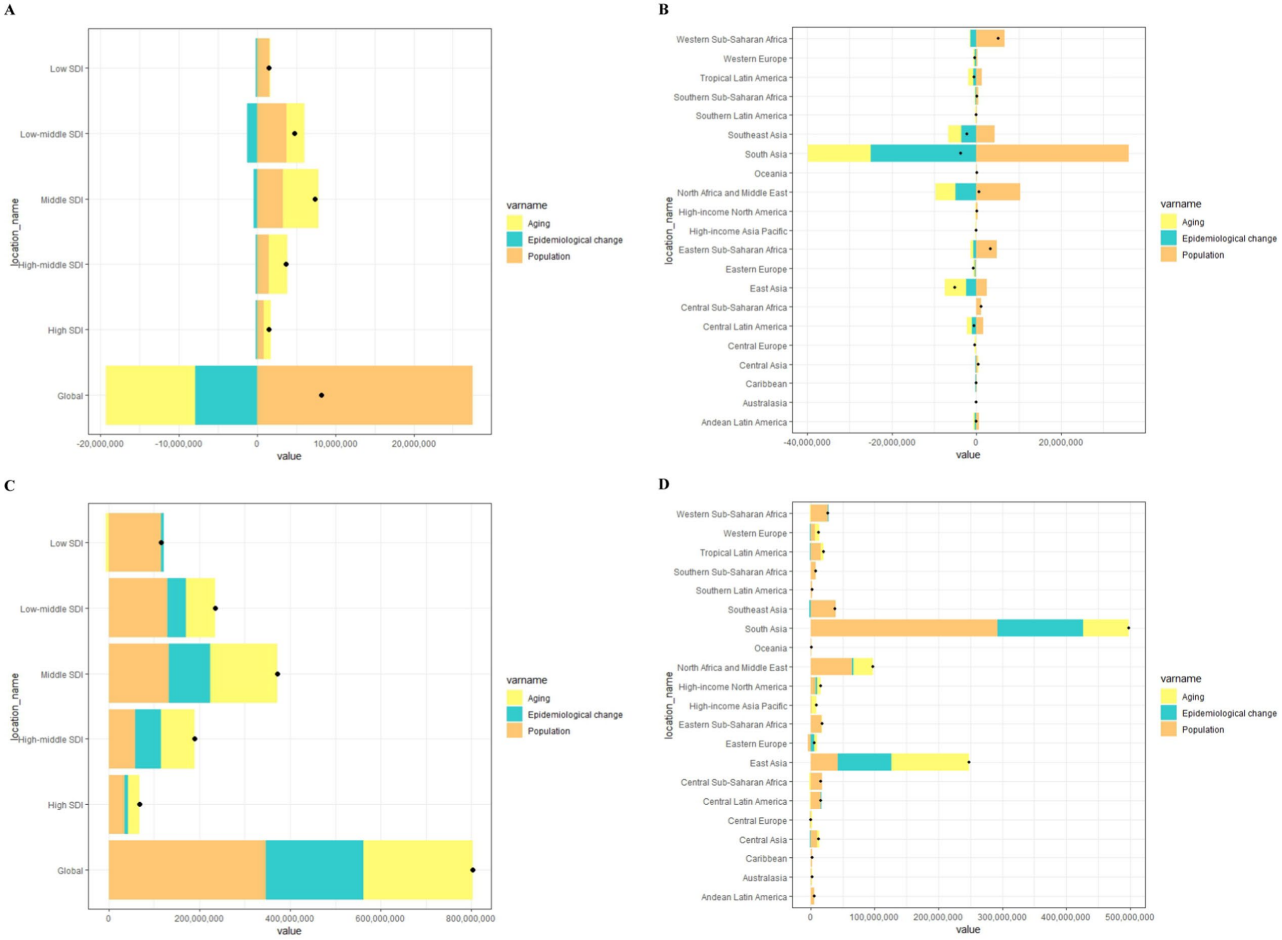
Changes in YLDs and prevalence of BVL according to ageing, population and epidemiological change from 1990 to 2021 at the global level by SDI quintile **(A,C)** and at the regional level **(B,D)**.

When stratified by SDI, aging had the least significant impact on YLDs and prevalence in low SDI countries, while population growth had the most substantial effect in these regions. Epidemiological changes had positive effects on prevalence but negative effects on YLDs. Regionally, South Asia and East Asia exhibited the most significant contributions of aging to the increase in YLDs, while population growth had the most significant impact in South Asia, and North Africa and the Middle East. The effects of epidemiological changes were most pronounced for both YLDs and prevalence in South Asia (Figure 2; Supplementary Table 2).

### Frontier analysis of ASYR

**Figure 3:**
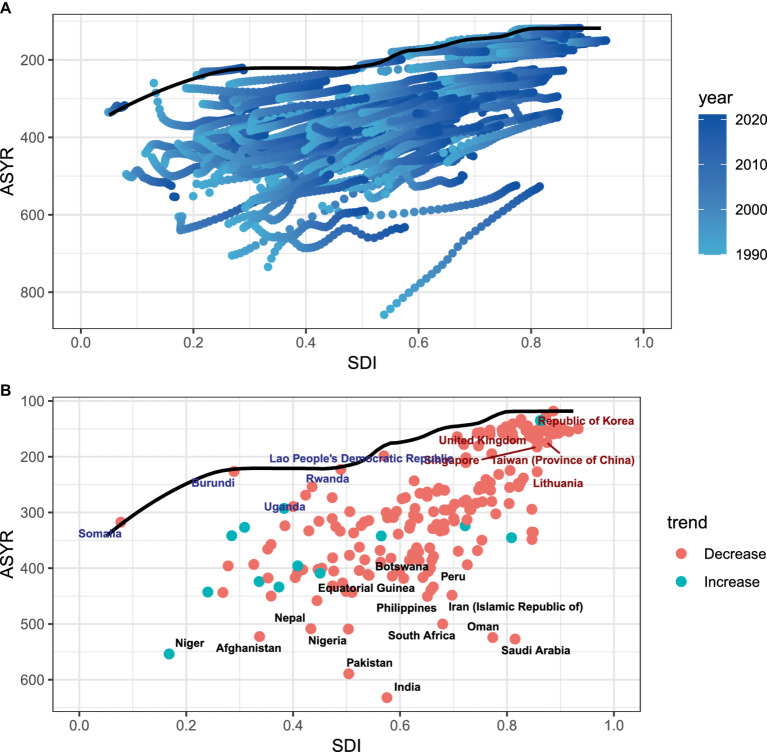
**(A,B)** Frontier analysis based on SDI and ASYR of BVL in 2021. The frontier is delineated in solid black color; countries and territories are represented as dots. The top 14 countries with the largest effective difference (largest BVL YLDs gap from the frontier) are labeled in black; examples of frontier countries with low SDI (<0.5) and low effective difference are labeled in blue (e.g., Somalia, Uganda, and Rwanda), and examples of countries and territories with high SDI (>0.85) and relatively high effective difference for their level of development are labeled in red (e.g., UK, Singapore and Korea. Red dots indicate a decrease in ASYR due to BVL from 1990 to 2021; green dots indicate an increase in ASYR due to BVL from 1990 to 2021.

Figure 3A shows the potential health improvements that countries and regions have missed over the past few decades based on different developmental stages. Figure 3B illustrates the YLDs for BVL and the health disparities among countries with varying levels of sociodemographic development in 2021. The effective difference in YLDs grows as sociodemographic development advances, suggesting that countries or regions with higher SDI have greater potential for health improvements.

### Health inequalities analysis

**Figure 4:**
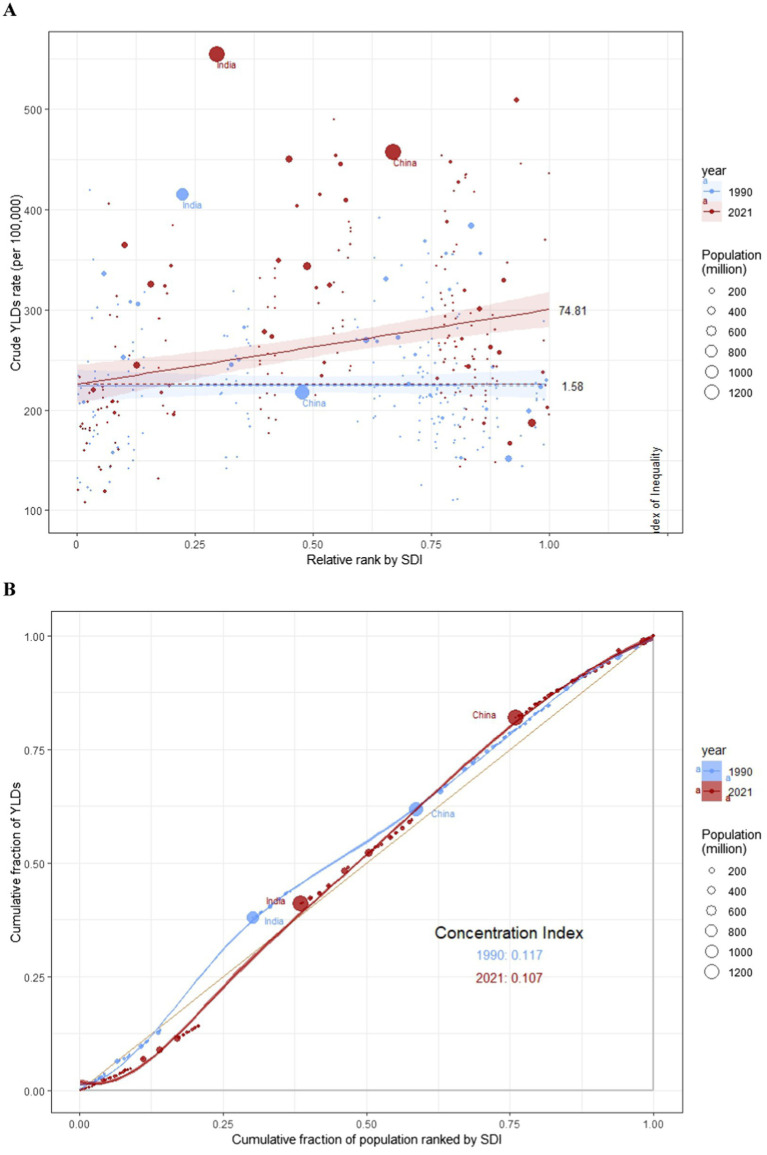
SII analysis and concentration index analysis. **(A)** Absolute income-related healthy inequality in BVL burden, presented using regression lines, 1990 vs 2021. **(B)** Relative income-related healthy inequality in BVL burden, presented using concentration curves, 1990 vs 2021.

The Slope Index of Inequality (SII) for YLDs was 1.58 (95% CI: −24.28 to 27.45) in 1990 and 74.81 (95% CI: 40.19–109.42) in 2021, indicating an upward trend in the association between ASYR and SDI, as shown in Figure 4A. This significant increase points to a growing inequality in the burden of BVL between economically prosperous and disadvantaged countries. Between 1990 and 2021, the Concentration Index for YLDs declined, suggesting a narrowing gap in BVL burden between richer and poorer countries. However, disparities in BVL burden persist, highlighting that, despite some narrowing of the wealth gap, global inequalities remain a major issue (Figure 4B).

### BAPC analysis

**Figure 5:**
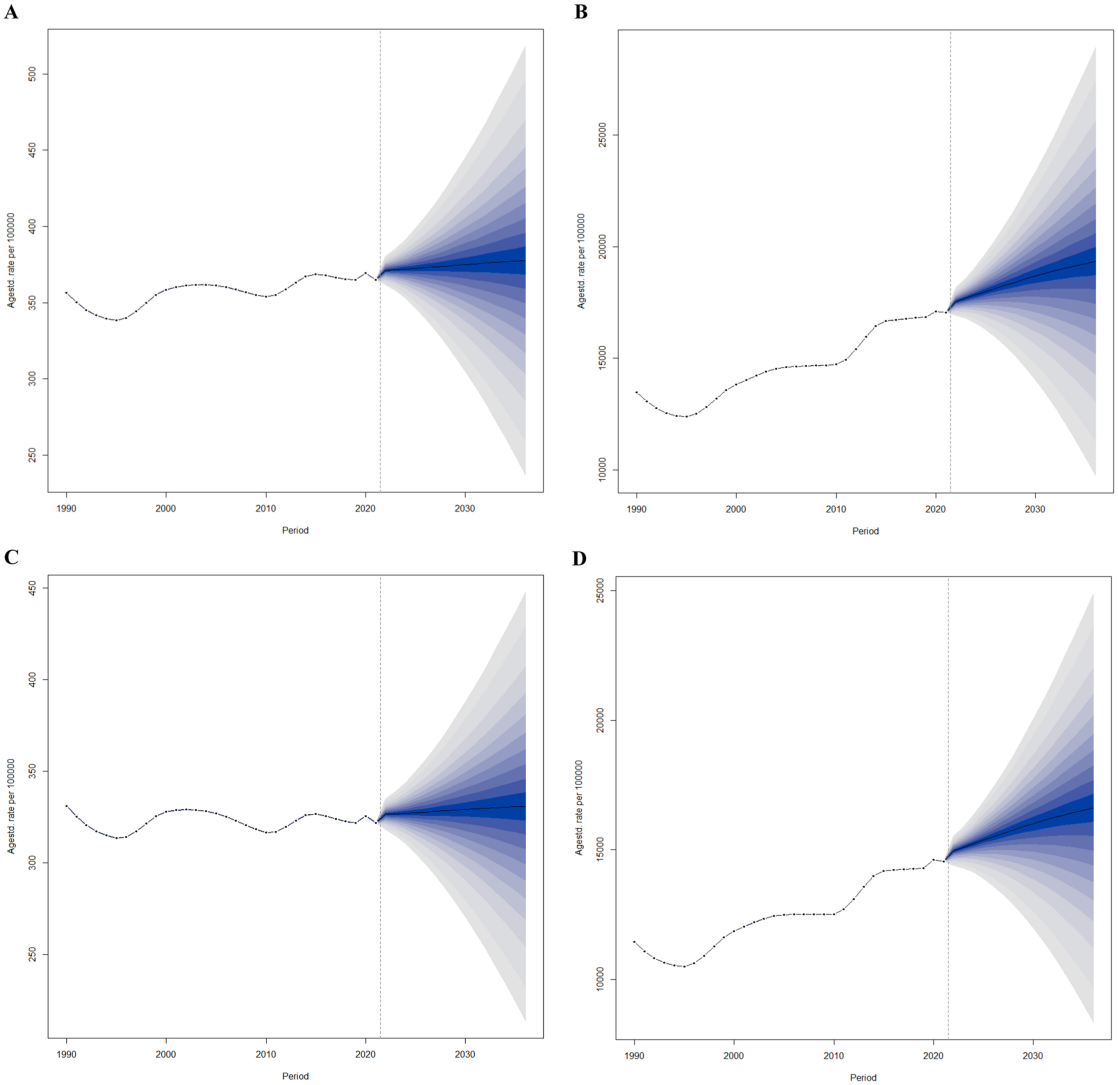
Trends of ASYR and ASPR of BVL in global from 2022 to 2036 in females **(A,B)** and males **(C,D)** predicted by Bayesian age-period-cohort models.

To project the future trends in ASYR and ASPR for BVL beyond 2021, we utilized BAPC models. As shown in Figure 5A, the ASYR for females is expected to increase annually from 371.21 per 100,000 in 2022 to 377.57 per 100,000 in 2036. Similarly, the ASYR for males is projected to rise from 326.52 per 100,000 in 2022 to 330.67 per 100,000 in 2036 (Figure 5C). Figure 5B illustrates the predicted rise in ASPR for females, from 17,537 per 100,000 in 2022 to 19,340 per 100,000 in 2036. Likewise, the ASPR for males is projected to increase from 14,959 per 100,000 in 2022 to 16,617 per 100,000 in 2036 (Figure 5D).

## Discussion

Our analysis, based on the latest GBD 2021 data, offers an unprecedented view into the developments in YLDs, the number of cases, ASYR, and ASPR of BVL globally between 1990 and 2021. The results show a significant increase in the global burden of BVL over the past 32 years, with both overall YLDs and the number of cases continuing to grow. Furthermore, projections for the next 15 years indicate that the burden is expected to rise, driven by population aging and changing epidemiological trends.

Cataracts, glaucoma, diabetic retinopathy, and refractive errors remain the leading causes of blindness and visual impairment (14). Research indicates that about 80% of cases of treatable BVL can be effectively prevented through early diagnosis and timely intervention, thus avoiding visual impairment (15). For example, the United States, a developed country, spent a staggering $134.2 billion in 2017 on economic expenditures related to vision loss (16). Given the magnitude of this issue, timely and accurate assessments of the BVL burden are crucial for countries to allocate healthcare resources effectively and plan public health strategies.

Since 1990, the global number of visually impaired people has shown considerable growth, increasing from 547 million in 1990 to 1.350 billion in 2021. This suggests a clear upward trend in the global burden of BVL over the last three decades. One of the primary factors driving this increase is the dramatic population expansion, which grew from 5.35 billion in 1990 to 7.89 billion in 2021 (17). Even after accounting for population growth, the upward trend in visual impairment persists, primarily due to increased life expectancy and the aging of the population. Between 1990 and 2021, global life expectancy increased from 63.8 to 71.7 years, and the proportion of the older adult increased from 8.5 to 12.1%. This demographic shift means that older populations are living with vision-threatening conditions for longer periods (6). Age is thus a significant risk factor for visual impairments (18).

Despite the increase in overall numbers, age-standardized measures of the global burden of visual impairment have gradually stabilized since the 1990s. This stabilization is largely attributed to sustained global socioeconomic development and significant improvements in healthcare services. However, some countries, including the United States, Russia, and China, have experienced noticeable increases in visual impairment. While population growth and aging are contributing factors, other elements such as urbanization, increased screen exposure, rising prevalence of diabetes, and disparities in healthcare access may also play a role. According to relevant reports from the United Nations, countries with the most rapidly aging populations include Japan, Italy, and Germany, whereas nations such as India and Nigeria exhibit the highest population growth rates (19). Therefore, further investigation is needed to fully understand the specific drivers of increasing visual impairment in these countries. Moreover, BVL continues to be a growing public health concern, particularly in the post-COVID-19 era, where factors like increased poverty and reduced access to healthcare services may further exacerbate this burden (20).

Our analysis reveals a notable gender disparity in the global burden of BVL, with both men and women experiencing an increase from 1990 to 2021. However, this rise has been more significant in females, who, in 2021, exhibited higher YLDs, a greater number of cases, and elevated ASYR and ASPR compared to males. Previous studies have demonstrated that gender influences the prevalence of various ocular diseases, such as diabetic retinopathy, age-related macular degeneration, and cataracts (21–23). The disparity stems from a complex interaction of biological, social, and economic factors. A major contributor is women’s greater life expectancy, which heightens their susceptibility to age-related visual impairments (24). Hormonal variations, particularly differences in estrogen levels, are also implicated in cataract formation (25). Beyond cataracts, other leading causes of blindness exhibit gender-specific trends. For instance, primary open-angle glaucoma is more prevalent in men, whereas women are more prone to primary angle-closure glaucoma, likely due to anatomical differences in anterior chamber depth (26). Additionally, diabetic retinopathy appears more frequently in men, possibly linked to variations in glycemic control and metabolic regulation (27). Conversely, women are at higher risk for age-related macular degeneration, which may be associated with longevity and hormonal changes affecting retinal health (28). Beyond biological determinants, disparities in healthcare access further exacerbate the gender gap in BVL. Women often encounter greater obstacles in obtaining eye care services, influenced by disparities in education, economic independence, and healthcare accessibility. These challenges contribute to a disproportionate burden of visual impairment among females (20). Addressing these disparities necessitates targeted interventions that consider both biological susceptibility and socioeconomic barriers.

This finding underscores the importance of addressing gender differences in eye health. Governments must prioritize building targeted monitoring and management systems for women’s eye health, ensuring greater access to eye care services. Improving eye health for women is essential for reducing the prevalence of visual impairment and promoting equitable health outcomes in society.

Our analysis reveals a strong correlation between BVL burden and socioeconomic indicators (2). Countries in the middle and lower-middle SDI quintiles have made significant strides in reducing the BVL burden, likely due to rapid development and the support of global initiatives like Vision 2020 and Universal Eye Health, advocated by the WHO (29). In contrast, countries with higher SDI levels, such as the US and Russia, face persistent challenges related to both population growth and aging, which could increase the BVL burden in the future.

The SII and CI in our study highlight a significant inverse relationship between BVL burden and socioeconomic status. Over the past three decades, the BVL burden has been disproportionately concentrated in economically disadvantaged countries. However, as disparities in wealth decrease globally, vision health challenges are still far from being equalized. This underlines the importance of international cooperation and proportional access strategies in addressing the unique eye health challenges faced by different regions, particularly in Africa and South Asia. Strengthening eye health systems in these regions is crucial for reducing global inequalities in vision health (30).

This study offers a comprehensive analysis of the spatial and temporal patterns of the BVL burden, drawing on the latest GBD 2021 data and a standardized methodology. Additionally, our forecasts for the future trends in the global BVL burden provide valuable insights for policymakers. However, there are some limitations to consider. First, the data and statistical assumptions inherent in the GBD methodology may introduce biases into the model estimates. Second, in remote or rural regions, where access to healthcare is limited, epidemiological data may be incomplete, potentially leading to an underestimation of the true BVL burden.

## Conclusion

In summary, the global burden of BVL has increased in the last 32 years, especially in high- and middle-income nations. This trend will continue over the next 15 years, influenced by demographic changes. Blindness and vision loss are major public health problems, and are more prominent among women and in low SDI regions. There is a global need to rationalize the allocation of resources, optimize health services and strengthen the prevention of BVL damage over the next 15 years.

## Data Availability

The original contributions presented in the study are included in the article/Supplementary material, further inquiries can be directed to the corresponding author.

## Footnotes

^1^ http://ghdx.healthdata.org/gbd-results-tool

## References

[1] GBD 2019 Blindness and Vision Impairment Collaborators, Vision loss expert Group of the Global Burden of Disease Study. Trends in prevalence of blindness and distance and near vision impairment over 30 years: an analysis for the global burden of disease study. Lancet Glob Health. (2021) 9:e130–43. doi: 10.1016/S2214-109X(20)30425-3 33275950 PMC7820390

[2] Ung L Jonas JB Lietman TM Chodosh J. COVID-19 and the unfinished agenda of VISION 2020. Am J Ophthalmol. (2021) 224:30–5. doi: 10.1016/j.ajo.2020.11.016, PMID: 33309690 PMC7831771

[3] Zhao C Ding Q Yang Z. Burdens and trends of blindness and vision loss among those aged 55 years and older: a systematic analysis for the global burden of disease study 2019. Eur J Ophthalmol. (2024) 34:1852–64. doi: 10.1177/11206721241238878, PMID: 38454852

[4] Noren Hooten N Pacheco NL Smith JT Evans MK. The accelerated aging phenotype: the role of race and social determinants of health on aging. Ageing Res Rev. (2022) 73:101536. doi: 10.1016/j.arr.2021.101536, PMID: 34883202 PMC10862389

[5] Truesdale BC Jencks C. The health effects of income inequality: averages and disparities. Annu Rev Public Health. (2016) 37:413–30. doi: 10.1146/annurev-publhealth-032315-021606 26735427

[6] Li Y Wang H Guan Z Guo C Guo P du Y. Persistence of severe global inequalities in the burden of blindness and vision loss from 1990 to 2019: findings from the global burden of disease study 2019. Br J Ophthalmol. (2024) 108:301–9. doi: 10.1136/bjo-2022-321801, PMID: 37423644

[7] GBD 2019 Blindness and Vision Impairment Collaborators. Causes of blindness and vision impairment in 2020 and trends over 30 years, and prevalence of avoidable blindness in relation to VISION 2020: the right to sight: an analysis for the global burden of disease study. Lancet Glob Health. (2021) 9:e144–60. doi: 10.1016/S2214-109X(20)30489-7 33275949 PMC7820391

[8] Jiang B Yao Q Yuan X Liu G Lu P. Burden of blindness and vision loss in China over the past 30 years: findings and predictions based on the global burden of disease study 2019. Br J Ophthalmol. (2024) 108:889–96. doi: 10.1136/bjo-2023-323527, PMID: 37474257

[9] Jiang B Jiang C Li J Lu P. Trends and disparities in disease burden of age-related macular degeneration from 1990 to 2019: results from the global burden of disease study 2019. Front Public Health. (2023) 17:1138428. doi: 10.3389/fpubh.2023.1138428 PMC10231224 37265519

[10] Wang J Rahman A Siegal HA Fisher JH. Standardization and decomposition of rates: useful analytic techniques for behavior and health studies. Behav Res Methods Instrum Comput. (2000) 32:357–66. doi: 10.3758/bf03207806 10875185

[11] GBD 2015 Healthcare Access and Quality Collaborators. Healthcare access and quality index based on mortality from causes amenable to personal health care in 195 countries and territories, 1990-2015: a novel analysis from the global burden of disease study 2015. Lancet. (2017) 390:231–66. doi: 10.1016/S0140-6736(17)30818-8 28528753 PMC5528124

[12] Hosseinpoor AR Bergen N Schlotheuber A. Promoting health equity: WHO health inequality monitoring at global and national levels. Glob Health Action. (2015) 8:29034. doi: 10.3402/gha.v8.29034, PMID: 26387506 PMC4576419

[13] Cai Y Zhang J Liang J Xiao M Zhang G Jing Z. The burden of rheumatoid arthritis: findings from the 2019 global burden of diseases study and forecasts for 2030 by Bayesian age-period-cohort analysis. J Clin Med. (2023) 12:1291. doi: 10.3390/jcm12041291 36835827 PMC9959633

[14] Bourne RRA Jonas JB Bron AM Cicinelli MV das A Flaxman SR. Prevalence and causes of vision loss in high-income countries and in eastern and Central Europe in 2015: magnitude, temporal trends and projections. Br J Ophthalmol. (2018) 102:575–85. doi: 10.1136/bjophthalmol-2017-311258, PMID: 29545417 PMC5909755

[15] Resnikoff S Pararajasegaram R. Blindness prevention programmes: past, present, and future. Bull World Health Organ. (2001) 79:222–6. PMID: 11285666 PMC2566377

[16] Rein DB Wittenborn JS Zhang P Sublett F Lamuda PA Lundeen EA. The economic burden of vision loss and blindness in the United States. Ophthalmology. (2022) 129:369–78. doi: 10.1016/j.ophtha.2021.09.010, PMID: 34560128

[17] GBD 2019 Demographics Collaborators. Global age-sex-specific fertility, mortality, healthy life expectancy (HALE), and population estimates in 204 countries and territories, 1950-2019: a comprehensive demographic analysis for the global burden of disease study 2019. Lancet. (2020) 396:1160–203. doi: 10.1016/S0140-6736(20)30977-6 33069325 PMC7566045

[18] Swenor BK Ehrlich JR. Ageing and vision loss: looking to the future. Lancet Glob Health. (2021) 9:e385–6. doi: 10.1016/S2214-109X(21)00031-0 33607013

[19] Shan W Xiu C Ji R. Creating a healthy environment for elderly people in urban public activity space. Int J Environ Res Public Health. (2020) 17:7301. doi: 10.3390/ijerph17197301, PMID: 33036270 PMC7579163

[20] Burton MJ Ramke J Marques AP Bourne RRA Congdon N Jones I. The lancet Global Health Commission on global eye health: vision beyond 2020. Lancet Glob Health. (2021) 9:e489–551. doi: 10.1016/S2214-109X(20)30488-5, PMID: 33607016 PMC7966694

[21] Lou L Wang J Xu P Ye X Ye J. Socioeconomic disparity in global burden of cataract: an analysis for 2013 with time trends since 1990. Am J Ophthalmol. (2017) 180:91–6. doi: 10.1016/j.ajo.2017.04.008, PMID: 28428050

[22] Lin X Lou L Miao Q Wang Y Jin K Shan P. The pattern and gender disparity in global burden of age-related macular degeneration. Eur J Ophthalmol. (2021) 31:1161–70. doi: 10.1177/1120672120927256, PMID: 32498618

[23] Xu Y Wang A Lin X Xu J Shan Y Pan X. Global burden and gender disparity of vision loss associated with diabetes retinopathy. Acta Ophthalmol. (2021) 99:431–40. doi: 10.1111/aos.14644, PMID: 33124190

[24] Prasad M Malhotra S Kalaivani M Vashist P Gupta SK. Gender differences in blindness, cataract blindness and cataract surgical coverage in India: a systematic review and meta-analysis. Br J Ophthalmol. (2020) 104:220–4. doi: 10.1136/bjophthalmol-2018-313562, PMID: 31221669

[25] Zetterberg M Celojevic D. Gender and cataract--the role of estrogen. Curr Eye Res. (2015) 40:176–90. doi: 10.3109/02713683.2014.898774, PMID: 24987869

[26] Kapetanakis VV Chan MP Foster PJ Cook DG Owen CG Rudnicka AR. Global variations and time trends in the prevalence of primary open angle glaucoma (POAG): a systematic review and meta-analysis. Br J Ophthalmol. (2016) 100:86–93. doi: 10.1136/bjophthalmol-2015-307223, PMID: 26286821 PMC4717368

[27] Wong TY Cheung CM Larsen M Sharma S Simó R. Diabetic retinopathy. Nat Rev Dis Primers. (2016) 2:16012. doi: 10.1038/nrdp.2016.12, PMID: 27159554

[28] Rudnicka AR Jarrar Z Wormald R Cook DG Fletcher A Owen CG. Age and gender variations in age-related macular degeneration prevalence in populations of European ancestry: a meta-analysis. Ophthalmology. (2012) 119:571–80. doi: 10.1016/j.ophtha.2011.09.027, PMID: 22176800

[29] Wang W Yan W Müller A Keel S He M. Association of Socioeconomics with prevalence of visual impairment and blindness. JAMA Ophthalmol. (2017) 135:1295–302. doi: 10.1001/jamaophthalmol.2017.3449, PMID: 29049446 PMC6583541

[30] Mwangi N Zondervan M Bascaran C. Analysis of an international collaboration for capacity building of human resources for eye care: case study of the college-college VISION 2020 LINK. Hum Resour Health. (2017) 15:22. doi: 10.1186/s12960-017-0196-1, PMID: 28288650 PMC5348790

