# Supplementary material for: An analysis of the global, regional, and national burden of blindness and vision loss between 1990 and 2021: the findings of the Global Burden of Disease Study 2021
Source: Front Public Health. 2025 Apr 29;13:1560449. doi: 10.3389/fpubh.2025.1560449 (PMC12070801; doi:10.3389/fpubh.2025.1560449)
Supplement: Supplementary file 1 [file Supplementary_file_1.docx]

**Supplementary Table 1** Prevalence of BVL in 1990 and 2021 for both sexes and all locations, with EAPC from 1990 to 2021.

| **Location** | | **1990** | |  | | |  | **2021** | |  | | **1990-2021** | |
| --- | --- | --- | --- | --- | --- | --- | --- | --- | --- | --- | --- | --- | --- |
|  | | **Cases No.×10^6^ (95%UI)** | | **ASPR No.×10^4^ (95%UI)** | | |  | **Cases No.×10^6^ (95%UI)** | | **ASPR No.×10^4^ (95%UI)** | | **EAPC (95%CI)** | |
| **Global** | | 547.02 (451.54,667.60) | | 1.25 (1.03,1.52) | | |  | 1350.05 (1087.46,1673.98) | | 1.58 (1.28,1.95) | | 1.09 (0.97 to 1.2) | |
| **Sex** | |  | |  | | |  |  | |  | |  | |
| Female | | 305.62 (251.74,372.42) | | 1.34 (1.11,1.65) | | |  | 746.24 (604.77,919.00) | | 1.70 (1.38,2.10) | | 1.09 (0.98 to 1.2) | |
| Male | | 241.40 (198.92,297.91) | | 1.14 (0.95,1.40) | | |  | 603.81 (482.18,755.43) | | 1.45 (1.17,1.80) | | 1.09 (0.97 to 1.21) | |
| **SDI** | |  | |  | | |  |  | |  | |  | |
| High SDI | | 81.86 (64.91,102.92) | | 0.81 (0.65,1.02) | | |  | 139.97 (111.05,176.06) | | 0.86 (0.69,1.09) | | 0.37 (0.32 to 0.42) | |
| High-middle SDI | | 119.24 (96.87,146.67) | | 1.16 (0.95,1.42) | | |  | 271.63 (214.67,341.46) | | 1.49 (1.19,1.87) | | 1.09 (0.97 to 1.21) | |
| Middle SDI | | 174.48 (143.84,213.55) | | 1.41 (1.16,1.70) | | |  | 495.40 (395.98,617.93) | | 1.79 (1.45,2.21) | | 1.11 (0.99 to 1.23) | |
| Low-middle SDI | | 124.62 (104.15,150.76) | | 1.67 (1.41,2.02) | | |  | 319.48 (259.78,393.41) | | 1.94 (1.60,2.36) | | 0.9 (0.76 to 1.04) | |
| Low SDI | | 46.35 (37.97,57.92) | | 1.59 (1.31,1.94) | | |  | 122.78 (99.38,153.25) | | 1.79 (1.47,2.17) | | 0.68 (0.58 to 0.78) | |
| **Region** | |  | |  | | |  |  | |  | |  | |
| Andean Latin America | | 4.32 (3.53,5.31) | | 1.69 (1.39,2.10) | | |  | 10.36 (8.36,12.92) | | 1.65 (1.34,2.04) | | -0.06 (-0.08 to -0.05) | |
| Australasia | | 1.80 (1.41,2.29) | | 0.82 (0.65,1.05) | | |  | 3.40 (2.68,4.29) | | 0.81 (0.65,1.04) | | -0.01 (-0.02 to 0) | |
| Caribbean | | 3.98 (3.26,4.89) | | 1.38 (1.12,1.70) | | |  | 7.04 (5.66,8.79) | | 1.35 (1.09,1.68) | | -0.03 (-0.05 to 0) | |
| Central Asia | | 6.00 (5.09,7.06) | | 1.17 (0.99,1.39) | | |  | 9.80 (8.06,11.96) | | 1.13 (0.94,1.37) | | -0.02 (-0.05 to 0.01) | |
| Central Europe | | 11.30 (9.16,14.07) | | 0.79 (0.64,0.98) | | |  | 14.66 (11.67,18.58) | | 0.78 (0.63,0.97) | | -0.02 (-0.04 to 0) | |
| Central Latin America | | 14.76 (12.56,17.43) | | 1.34 (1.14,1.57) | | |  | 37.32 (30.26,46.38) | | 1.45 (1.18,1.78) | | 0.5 (0.39 to 0.61) | |
| Central Sub-Saharan Africa | | 6.49 (4.95,8.52) | | 1.96 (1.54,2.52) | | |  | 16.86 (12.83,22.50) | | 1.95 (1.53,2.50) | | 0 (-0.01 to 0) | |
| East Asia | 118.32 (94.61,148.02) | | 1.21 (0.98,1.51) | |  | 366.36 (281.82,472.98) | | | 1.73 (1.35,2.20) | | 1.4 (1.25 to 1.56) | |  |
| Eastern Europe | | 36.73 (29.71,45.42) | | 1.38 (1.13,1.69) | | |  | 48.98 (38.95,60.75) | | 1.60 (1.29,1.99) | | 1.15 (0.91 to 1.38) | |
| Eastern Sub-Saharan Africa | | 13.25 (10.69,16.55) | | 1.30 (1.06,1.59) | | |  | 31.26 (24.98,39.67) | | 1.29 (1.05,1.58) | | 0.08 (0.04 to 0.13) | |
| High-income Asia Pacific | | 16.79 (13.00,21.98) | | 0.84 (0.66,1.09) | | |  | 25.89 (20.36,32.87) | | 0.82 (0.65,1.07) | | -0.06 (-0.07 to -0.05) | |
| High-income North America | | 20.97 (16.56,26.56) | | 0.68 (0.53,0.87) | | |  | 36.45 (28.22,46.80) | | 0.73 (0.56,0.96) | | 0.76 (0.57 to 0.95) | |
| North Africa and Middle East | | 29.26 (25.11,33.77) | | 1.35 (1.15,1.56) | | |  | 72.71 (59.55,90.45) | | 1.37 (1.15,1.67) | | 0.07 (0.03 to 0.12) | |
| Oceania | | 0.53 (0.44,0.66) | | 1.41 (1.17,1.71) | | |  | 1.30 (1.06,1.62) | | 1.39 (1.15,1.70) | | -0.04 (-0.07 to -0.02) | |
| South Asia | | 128.12 (106.01,156.66) | | 1.83 (1.54,2.20) | | |  | 402.13 (319.62,496.50) | | 2.40 (1.94,2.92) | | 1.51 (1.29 to 1.72) | |
| Southeast Asia | | 41.87 (34.90,50.89) | | 1.34 (1.13,1.60) | | |  | 94.07 (76.45,116.54) | | 1.35 (1.12,1.64) | | 0.04 (0 to 0.09) | |
| Southern Latin America | | 4.40 (3.60,5.47) | | 0.93 (0.76,1.17) | | |  | 7.26 (5.89,9.13) | | 0.92 (0.74,1.15) | | -0.04 (-0.05 to -0.04) | |
| Southern Sub-Saharan Africa | | 9.49 (7.43,12.25) | | 2.59 (2.06,3.25) | | |  | 19.17 (14.97,24.30) | | 2.66 (2.14,3.30) | | 0.71 (0.52 to 0.9) | |
| Tropical Latin America | | 20.14 (16.38,25.23) | | 1.75 (1.42,2.16) | | |  | 43.81 (35.24,55.04) | | 1.72 (1.40,2.14) | | 0.21 (0.08 to 0.33) | |
| Western Europe | | 40.60 (32.76,50.94) | | 0.81 (0.66,1.01) | | |  | 56.17 (44.72,71.07) | | 0.79 (0.65,0.99) | | -0.05 (-0.07 to -0.03) | |
| Western Sub-Saharan Africa | | 17.88 (14.27,22.77) | | 1.56 (1.25,1.96) | | |  | 45.07 (36.16,57.85) | | 1.58 (1.29,1.96) | | 0.11 (0.08 to 0.14) | |

UI, uncertainty interval; CI, confidence interval; EAPC, estimated annual percentage change; SDI, socio-demographic index; ASPR, age-standardized prevalence rates.

**Supplementary Table 2** Changes in YLDs and prevalence number according to population-level determinants and causes from 1990 to 2021.

| **Location** | **Overll difference** | **Changes in YLDs number due to Population-level determinants** | | |  | **Overll**  **difference** | **Changes in prevalence number due to Population-level determinants** | | |
| --- | --- | --- | --- | --- | --- | --- | --- | --- | --- |
|  |  | **Aging** | **Population** | **epidemiological change** |  |  | **Aging** | **Population** | **epidemiological change** |
| Global | 8225638.97 | -11394062.74 | 27503985.15 | -7884283.441 |  | 803036908.5 | 241232397.4 | 346311729.1 | 215492782 |
| High SDI | 1475520.03 | 856258.446 | 832837.195 | -213575.615 |  | 67538659.5 | 24887837.71 | 35354999.25 | 7295822.554 |
| High-middle SDI | 3641930.06 | 2348431.901 | 1467643.463 | -174145.302 |  | 188979473.9 | 74091896.72 | 58911874.31 | 55975702.88 |
| Middle SDI | 7364811.36 | 4507206.017 | 3319073.767 | -461468.424 |  | 371070401.9 | 147560627.2 | 131800819 | 91708955.64 |
| Low-middle SDI | 4771935.58 | 2270734.337 | 3775292.787 | -1274091.542 |  | 235045795.4 | 64992227.72 | 128370490 | 41683077.73 |
| Low SDI | 1495594.22 | 77508.894 | 1561558.517 | -143473.194 |  | 115306169.9 | -5673539.068 | 115504800.9 | 5474908.052 |
| Andean Latin America | -52496.09 | -300753.92 | 598223.019 | -349965.184 |  | 5456594.11 | 172594.463 | 5643194.059 | -359194.416 |
| Australasia | 24018.08 | -9350.117 | 46086.284 | -12718.091 |  | 1777818.38 | 568431.094 | 1237265.793 | -27878.51 |
| Caribbean | -51603.05 | -99346.672 | 144908.965 | -97165.342 |  | 2330330.76 | 329482.062 | 2159151.539 | -158302.837 |
| Central Asia | 399678.56 | 235891.363 | 281599.647 | -117812.454 |  | 12434021.35 | 3161850.811 | 10189335.28 | -917164.748 |
| Central Europe | -308128.21 | -191614.13 | -52028.586 | -64485.493 |  | 415254.48 | 1895954.362 | -1249790.473 | -230909.406 |
| Central Latin America | -587683.26 | -1196632.277 | 1607507.439 | -998558.42 |  | 16049709.6 | -1053740.164 | 15967018.77 | 1136430.993 |
| Central Sub-Saharan Africa | 1186876.59 | -8565.204 | 1118924.379 | 76517.419 |  | 15825271.34 | -2029353.819 | 18117638.63 | -263013.468 |
| East Asia | -5052989.41 | -5163141.985 | 2483878.277 | -2373725.702 |  | 248217341.3 | 121581861.1 | 42774778.59 | 83860701.62 |
| Eastern Europe | -744320.67 | -356630.115 | -176545.86 | -211144.699 |  | 5832200.01 | 4123195.923 | -4188946.171 | 5897950.262 |
| Eastern Sub-Saharan Africa | 3409633.3 | -667459.874 | 4858781.905 | -781688.731 |  | 18011098.92 | 1313895.703 | 16803077.17 | -105873.953 |
| High-income Asia Pacific | -96034.49 | -90263.145 | 42166.473 | -47937.818 |  | 9105642.64 | 8104907.884 | 1428722.634 | -427987.876 |
| High-income North America | 165397.38 | -85153.282 | 308071.574 | -57520.914 |  | 15474390 | 5892225.917 | 7621738.502 | 1960425.586 |
| North Africa and Middle East | 625439.85 | -4806579.748 | 10348796.81 | -4916777.219 |  | 97600775.15 | 30399066.12 | 64740422.01 | 2461287.017 |
| Oceania | 93668.82 | -17087.456 | 125442.185 | -14685.905 |  | 1231673.25 | -12251.102 | 1312249.366 | -68325.009 |
| South Asia | -3725824.22 | -14817828.18 | 36067498.69 | -24975494.74 |  | 498278503.3 | 71160396.63 | 292314564.1 | 134803542.6 |
| Southeast Asia | -2172803.1 | -2969365.727 | 4359659.757 | -3563097.13 |  | 37656716.55 | 421130.753 | 39010734.13 | -1775148.326 |
| Southern Latin America | -31504.32 | -93924.052 | 136854.724 | -74434.994 |  | 1854975.96 | -193013.449 | 2267839.856 | -219850.451 |
| Southern Sub-Saharan Africa | 167630.89 | -212988.914 | 485031.589 | -104411.78 |  | 8213952.1 | 881831.735 | 7353146.639 | -21026.275 |
| Tropical Latin America | -617451.77 | -1126294.38 | 1280578.347 | -771735.74 |  | 19792100.82 | 5154598.68 | 15498081.46 | -860579.313 |
| Western Europe | -372620.26 | -319123.17 | 292143.398 | -345640.486 |  | 12158129.66 | 6709880.998 | 6698764.8 | -1250516.139 |
| Western Sub-Saharan Africa | 5210795.59 | -219796.138 | 6758773.368 | -1328181.641 |  | 27189992.84 | -676256.694 | 27376527.19 | 489722.343 |

**Supplementary Figure 1**

**
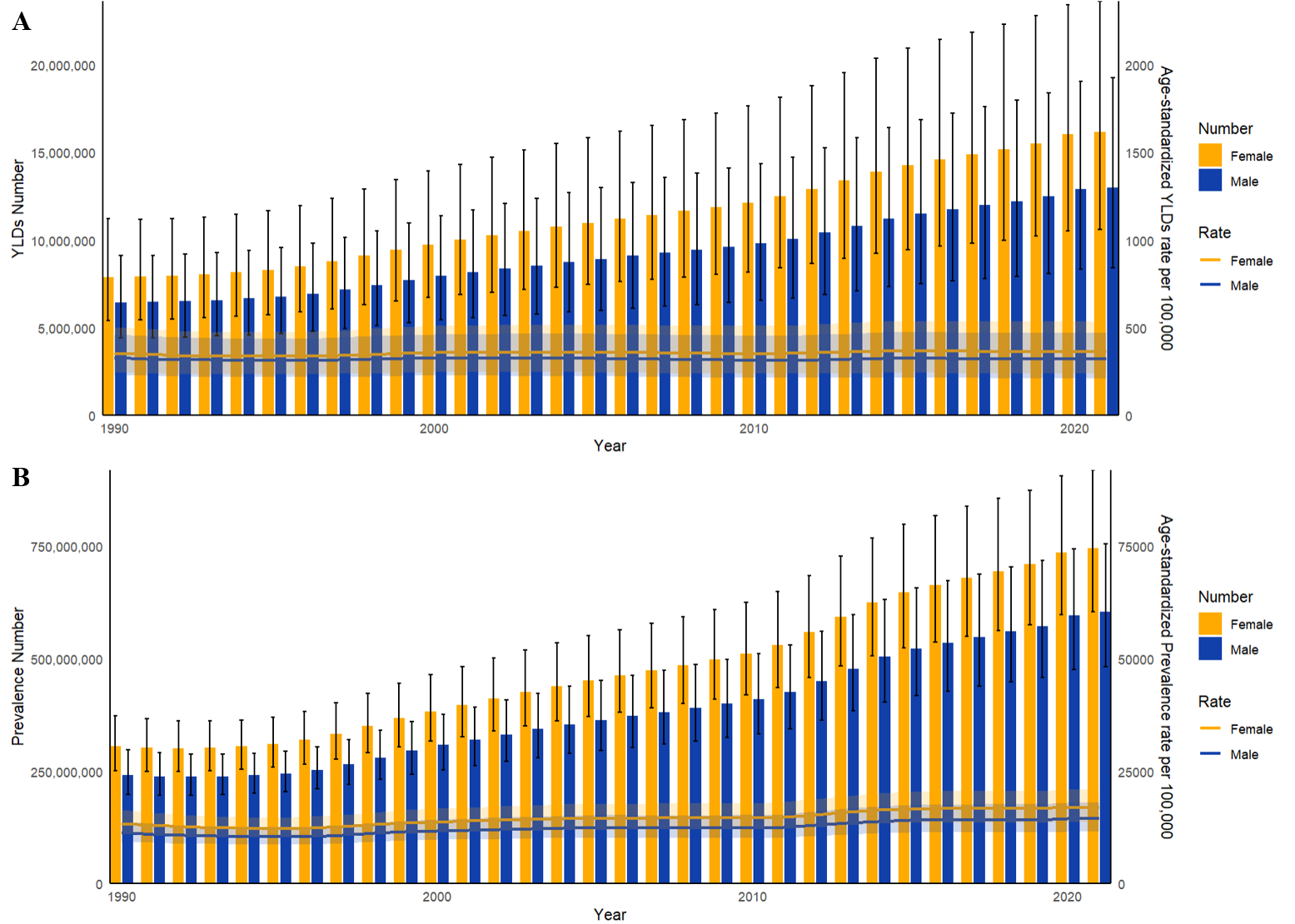
**

Trends in the all-age cases and age-standardized YLDs and prevalence rates of BVL by sex from 1990 to 2021. (A) YLDs number and rate. (B) Prevalence number and rate.

**Supplementary Figure 2**


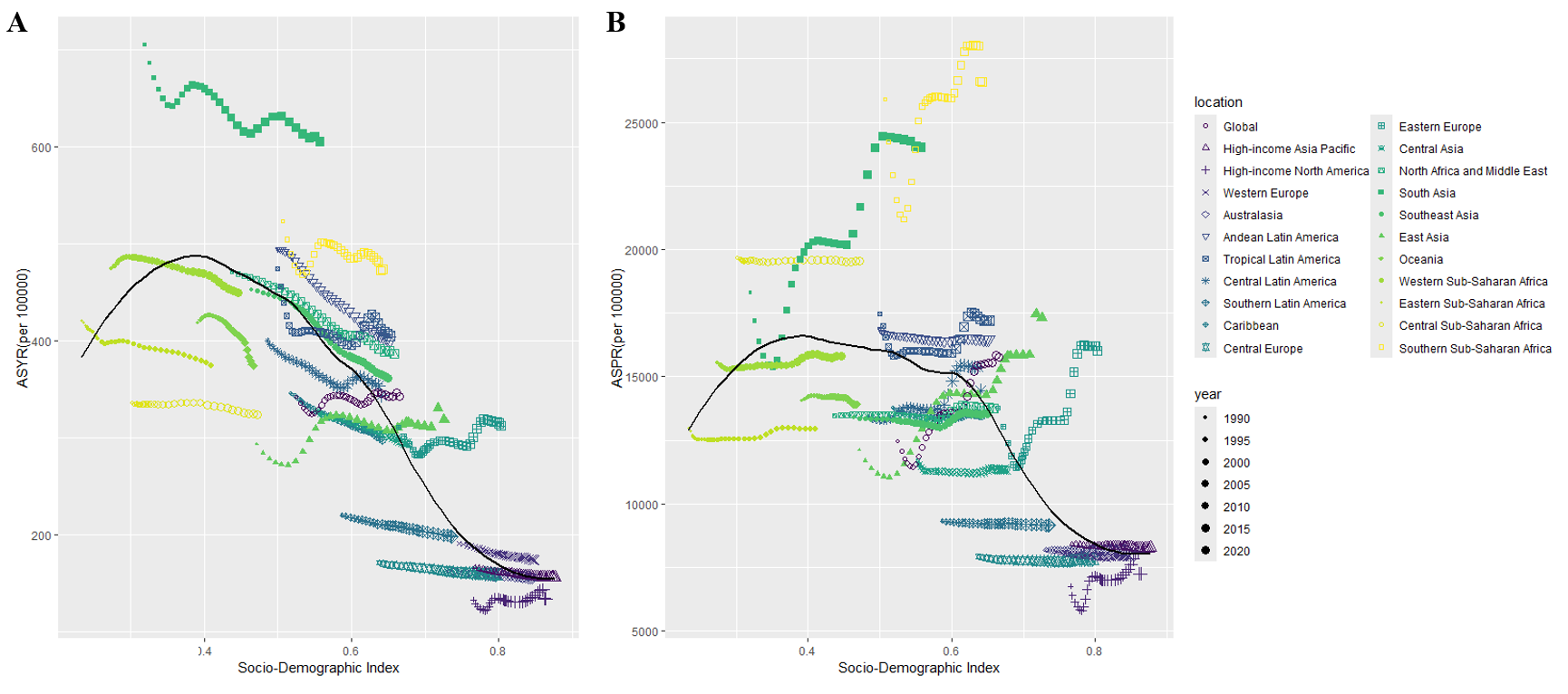


Age-standardized burden rate attributable to BVL across 21 GBD regions by socio-demographic index from 1990 to 2021. (A) ASYR; (B) ASPR. The black line was an adaptive association fitted with adaptive Loess regression based on all data points. For each region, points from left to right depict estimates from each year from 1990 to 2021.

**Supplementary Figure 3**


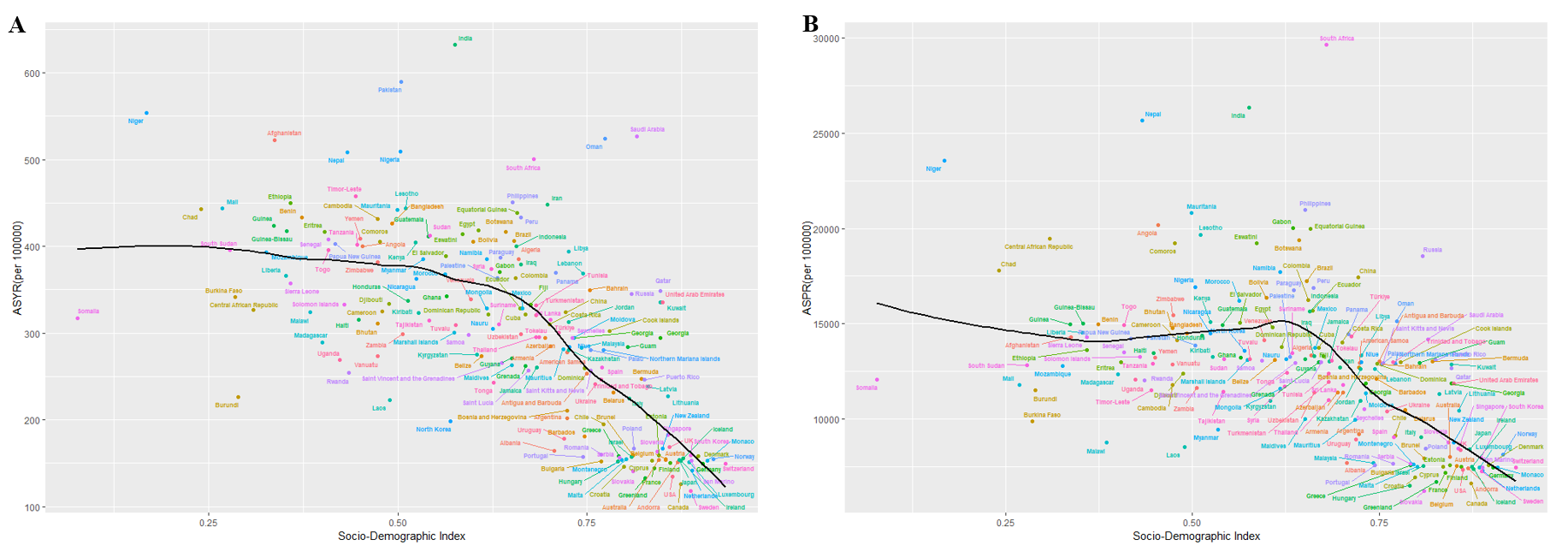


The factors associated with the ASYR(A) and ASPR(B) attributable to BVL at the national level in 2021. The black line was an adaptive association fitted with adaptive Loess regression based on all data points.
